# A Rare Case of Eosinophilic Gastroenteritis Isolated in the Muscularis Propria of the Small Bowel

**DOI:** 10.7759/cureus.18790

**Published:** 2021-10-14

**Authors:** Elina Kim, Varun Kesar, Douglas Grider, Vu Nguyen

**Affiliations:** 1 Gastroenterology, Virginia Tech Carilion School of Medicine, Roanoke, USA; 2 Gastroenterology, Carilion Roanoke Memorial Hospital, Roanoke, USA; 3 Pathology, Carilion Roanoke Memorial Hospital, Roanoke, USA; 4 Basic Science Education, Virginia Tech Carilion School of Medicine, Roanoke, USA; 5 Gastroenterology, University Hospitals Cleveland Medical Center, Cleveland, USA

**Keywords:** adult gastroenterology, eosinophilic gastroenteritis, eosinophilic gastroenteritis diagnosis, full-thickness biopsy, gastroenterology and endoscopy, muscular type eosinophilic gastroenteritis

## Abstract

Eosinophilic gastroenteritis (EG) is an autoimmune disorder that involves infiltration of eosinophils in the bowel wall of the stomach and/or intestine, resulting in various gastrointestinal symptoms. The majority of cases are diagnosed by findings of increased eosinophils on mucosal biopsies. We describe a rare type of eosinophilic gastroenteritis with eosinophilic infiltration involving only the muscularis propria layer. This elusive diagnosis was made after a full-thickness intestinal wall biopsy. This predominantly muscular type eosinophilic gastroenteritis can cause intestinal obstruction or perforation. Similar to the predominantly mucosal type eosinophilic gastroenteritis, this type of eosinophilic gastroenteritis responds to low-dose or topical corticosteroids.

## Introduction

Eosinophilic gastroenteritis (EG) involves eosinophilic infiltration of the bowel wall and presents with various gastrointestinal manifestations [1]. Three subtypes of EG exist based on the depth of eosinophilic invasion: mucosal, muscular, and serosal types. Mucosal type EG is the most common, with an estimated prevalence of 88%-100% [2,3], and muscular and serosal types usually present concomitantly with some mucosal eosinophilic infiltration. Thus, endoscopic biopsies are known to play an important role in diagnosis, with a detection rate of 80% [4]. However, in this study, we report a rare case of a 57-year-old male with muscular type EG that had isolated eosinophilic infiltration of the muscularis propria without mucosal involvement. This case highlights the importance of a thorough diagnostic workup to identify atypical variants of EG that may not be initially apparent on endoscopic biopsy.

## Case presentation

A 57-year-old male patient presented for follow-up of chronic abdominal pain. His past medical history was significant for hypertension, hyperlipidemia, chronic back pain, and allergic rhinitis. The patient has had episodes of intermittent abdominal pain and watery diarrhea for the past 10 years requiring numerous hospitalizations. Flares typically began with periumbilical cramping and then expanded to stabbing lower quadrant abdominal pain. Abdominal pain was associated with watery diarrhea sometimes, averaging over eight bowel movements per day. The patient denied the association of symptoms to food intake. His family history was unremarkable. Review of systems revealed rash and itching on the chest wall, nasal congestion, abdominal pain, abdominal distension, and diarrhea. Physical examination showed a soft, non-distended abdomen with suprapubic and left lower quadrant abdominal tenderness, guarding, and active bowel sounds.

**Figure 1:**
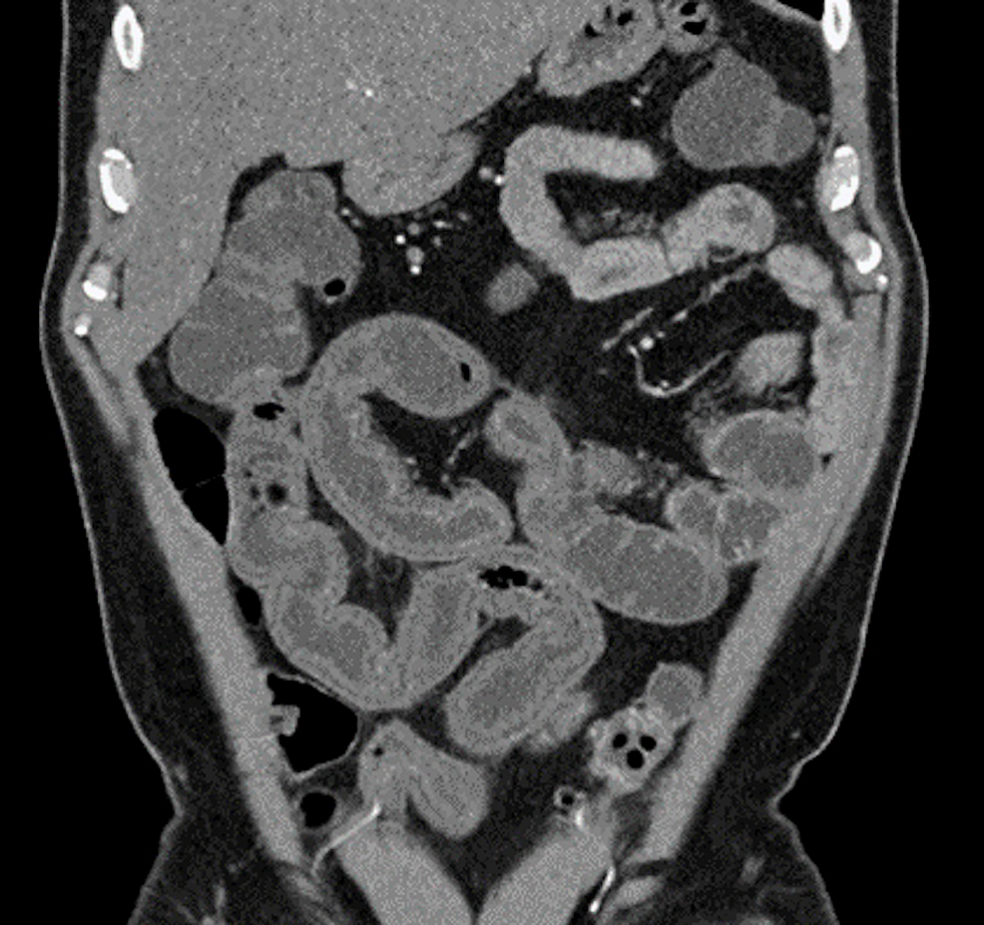
Computed Tomography Scan Computed tomography coronal view showed multiple loops of the small intestine with wall thickening, edema, and hyperenhancement.

**Figure 2:**
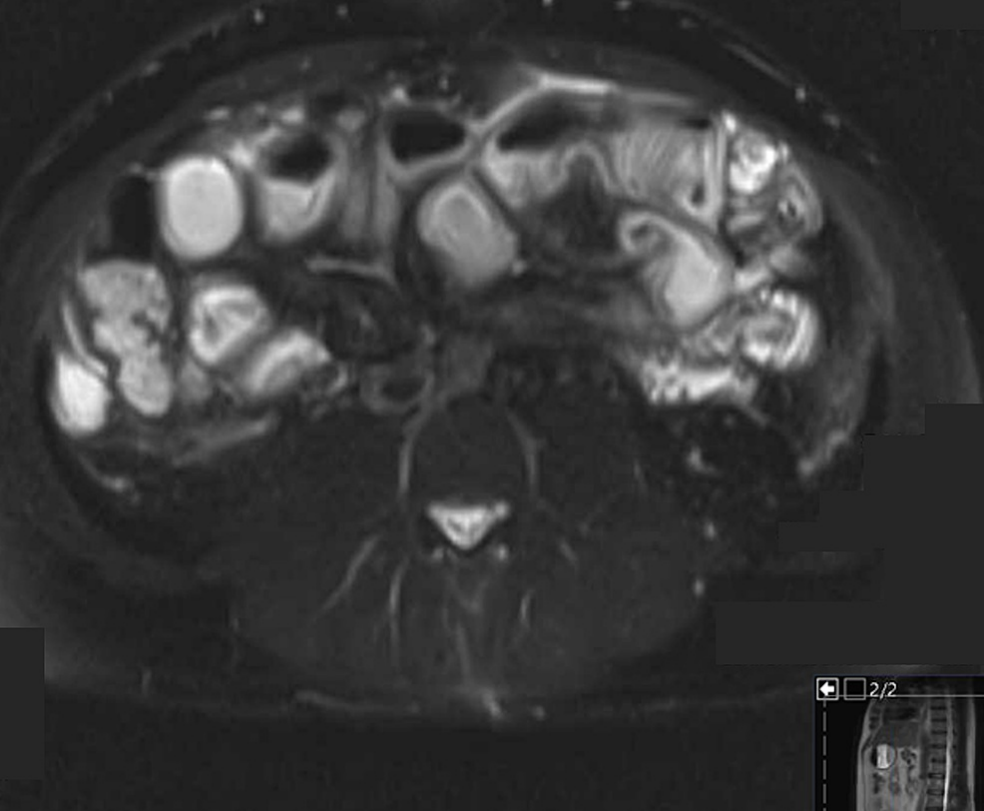
Magnetic Resonance Enterography Magnetic resonance enterography T2-weighted cross-sectional view of the abdomen and pelvis showed multiple loops of the small intestine with wall thickening, edema, and hyperenhancement.

Multiple CT scans and MR enterographies over the past 10 years showed multiple loops of the small intestine with wall thickening, edema, and hyperenhancement (Figures 1 and 2). Autoimmune and complement levels, including tissue transglutaminase IgA, antinuclear antibodies, antineutrophilic cytoplasmic antibodies, C1 esterase inhibitor, C3, and C4, were normal. The patient had elevated fecal lactoferrin of 87 μg/mL during one admission (normal range: <7.24 μg/mL), whereas other stool tests, including culture, *Clostridioides difficile* toxin, and ova and parasite examination, were normal. Urine porphobilinogen level was normal. Food allergy testing and alpha-gal testing were also normal. His symptoms usually subsided after several days of conservative management.

**Figure 3:**
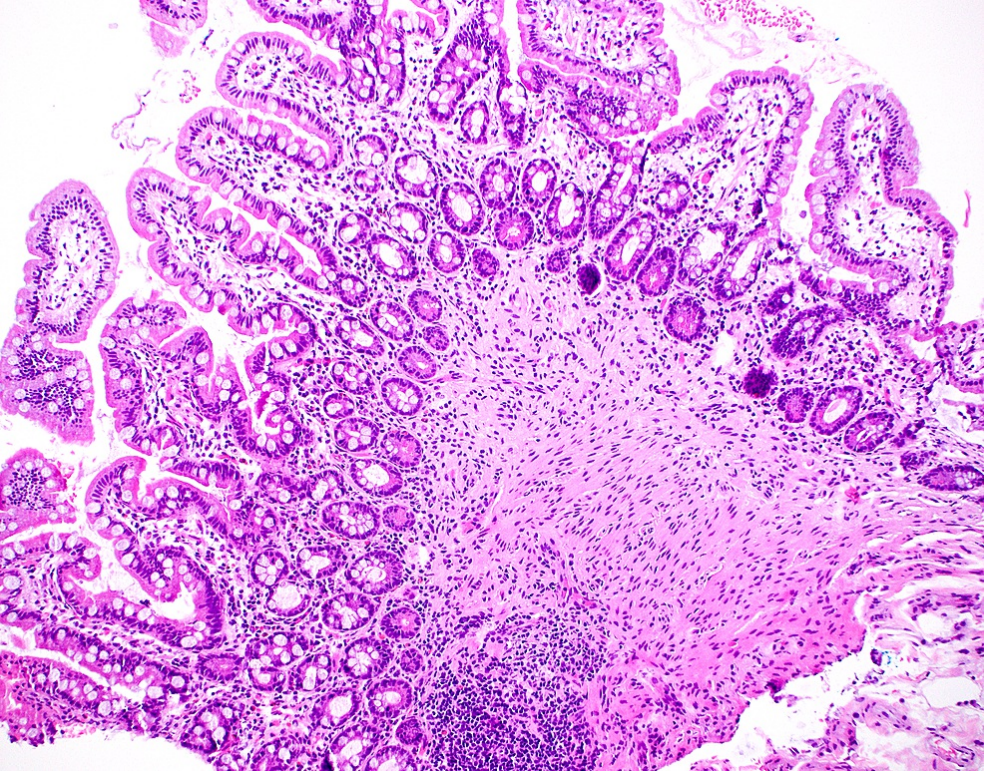
Mucosal Biopsy of the Small Bowel Hematoxylin and eosin stains at 100x magnification of the small bowel superficial layer (mucosa and muscularis mucosa) did not show increased eosinophils.

The patient previously had extensive diagnostic endoscopies, including several esophagogastroduodenoscopies, colonoscopies, capsule endoscopies, and single-balloon enteroscopy, for deep small intestine evaluation over the years. Mucosal biopsies from the stomach, small intestine (Figure 3), and colon showed no significant abnormality. One gastric biopsy report suggested a possible slight increase in mast cells, a controversial finding as there has been no established number of mast cells in any gastric or intestinal biopsy specimen. Although tryptase level was unremarkable and the diagnosis of mastocytosis was very unlikely, the patient was started on a course of cromolyn. As expected, this therapy provided minimal relief.

**Figure 4:**
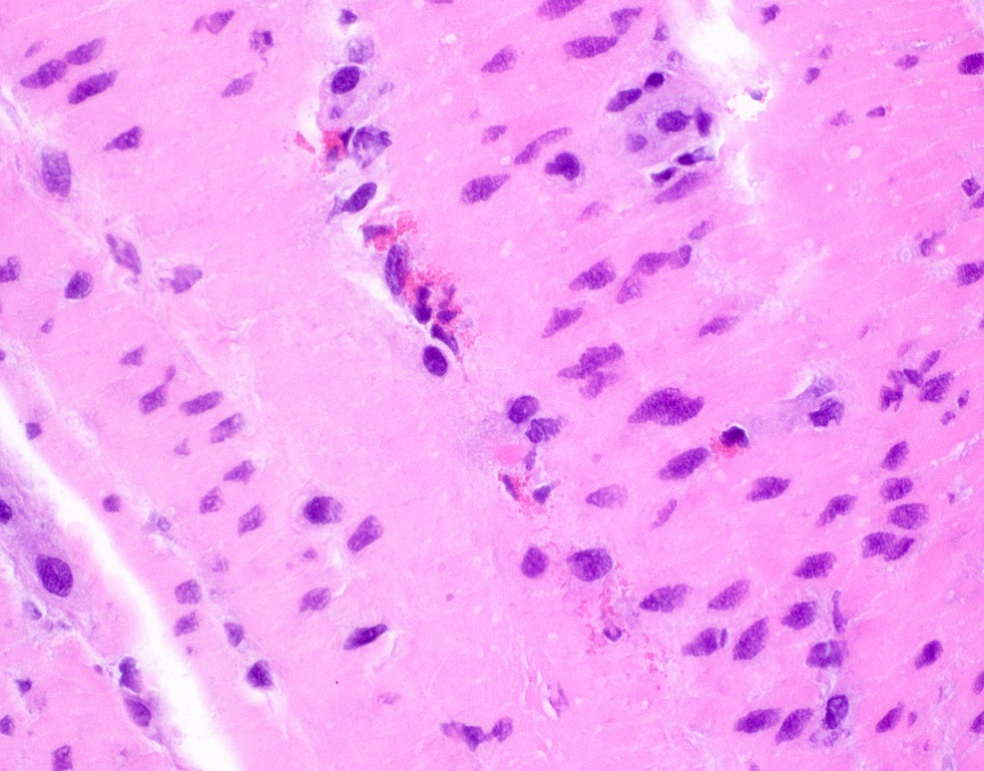
Full-Thickness Biopsy of the Small Bowel Hematoxylin and eosin stains at 600x magnification of the small bowel obtained from the full-thickness biopsy showed the presence of degranulating eosinophils in the muscularis propria layer.

Due to the patient’s continuing symptoms, he was referred for exploratory laparoscopy, which revealed small bowel edema and adhesions. Laparoscopic full-thickness small bowel biopsies revealed patchy increases in eosinophils predominantly in the muscularis propria, which were degranulating (Figure 4). Hence, a diagnosis of predominant muscular type eosinophilic gastroenteritis was established. Subsequently, the patient was started on budesonide 9 mg daily. When budesonide was later tapered to 6 mg, the patient experienced worsening of symptoms, so he was maintained on the 9 mg dose. The patient was already seeing an allergist who agreed with our treatment plan. On this regimen, the patient reported significant symptomatic relief with no recurrence of symptoms after over a year of follow-up.

## Discussion

First described by Kaijser in 1937, EG is a rare condition involving eosinophilic infiltration of the bowel wall [5]. The exact epidemiology of EG is unclear, but the US prevalence is estimated to range from 2.5/100,000 to 28/100,000 [6,7]. EG affects both children and adults and most commonly presents in the third to fifth decade of life. There is a slight male to female predominance of 1.2:1 [7].

The clinical manifestations of EG are highly variable and depend on the degree and location of eosinophilic infiltration. EG can manifest anywhere from the esophagus to the rectum, although the stomach and duodenum are most commonly involved [8,9]. Adults usually present with diarrhea, abdominal pain, weight loss, anemia, nausea, and vomiting. Approximately 80% of patients experience chronic waxing and waning abdominal pain [10]. Children present with failure to thrive, growth retardation, delayed puberty, and delayed menarche. Based on the predominant location of eosinophilic invasion, EG can be subdivided into mucosal, muscular, and serosal types. Mucosal type EG is the most common, with an estimated prevalence of 88%-100% [2,3], and can cause protein-losing enteropathy, bleeding, and malabsorption. Muscular type EG, as seen in our patient, is the second most common. It can cause intestinal obstruction and perforation and is the most likely type to relapse. Serosal type EG can present with ascites [11]. Fatalities are rare.

The etiology and pathophysiology of EG remain poorly understood. Studies suggest that an altered immune response to a food or environmental allergen may activate eosinophils to release inflammatory mediators [12,13]. Interestingly, many patients have associated allergy conditions such as asthma, eczema, rhinitis, and drug or food allergies. Furthermore, 64% of patients report a family history of atopic disease [14]. Our patient also reported allergic symptoms since his teen years, particularly to pollen and dust mites.

EG diagnosis requires eosinophilic infiltration of the bowel wall, exclusion of other causes of peripheral eosinophilia, and absence of parasitic disease. Radiologic changes are nonspecific, variable, or absent in 40% of cases [15]. CT scan may show irregular thickening of the stomach and small intestine. Endoscopic appearance is usually nonspecific, adding to the diagnostic challenge.

The detection rate of EG by endoscopic biopsy is approximately 80% [4]. However, as seen in our patient, a full-thickness laparoscopic biopsy may be required, particularly for the muscular and serosal types. Histopathology usually shows patchy eosinophilic infiltration. Although mucosal type EG often shows >50 eosinophils per high-power field [15], there is no defined diagnostic threshold when it comes to muscular type EG. Mast cells, submucosal edema, and loss of villi may also be seen [16,17]. Potential laboratory findings include peripheral eosinophilia, elevated IgE, iron-deficiency anemia, and low albumin [15], none of which were observed in our patient. Patients are usually responsive to low-dose or topical corticosteroids [4]. Mast cell stabilizers may also be used [18].

## Conclusions

We describe a rare type of eosinophilic gastroenteritis (EG) with eosinophilic infiltration involving only the muscularis propria layer. This case highlights the importance of full-thickness biopsy for diagnosing muscular type EG, specifically when endoscopy has been inconclusive. This requires multidisciplinary discussion involving the pathologist, gastroenterologist, and surgeon to make a timely diagnosis so that appropriate treatment can be initiated to prevent relapse and complications.
